# Otherwise Unexplained Transient QTc Prolongation in a Patient Admitted with COVID Disease

**DOI:** 10.1155/2021/9931405

**Published:** 2021-05-31

**Authors:** Mark Coyle, Mark Wilkinson, Mark Sheehy

**Affiliations:** Department of Medicine, Midlands Regional Hospital Mullingar, Longford Road, Robinstown, Mullingar, Westmeath, Ireland

## Abstract

*Background*. Several cardiovascular manifestations of coronavirus disease 2019 (COVID-19) have been previously described. QT prolongation has been reported in COVID-19 infection in association with medications such as azithromycin, hydroxychloroquine, and chloroquine but has not previously been reported as a direct result of COVID-19 infection. *Case summary*. We report the case of a 65-year-old female who developed a prolonged corrected QT interval (QTc) during a hospital admission with COVID-19. This patient was not on any QT prolonging treatment, serum electrolytes were normal, and there was no identifiable reversible cause for the QTc lengthening. Daily serial ECGs during admission showed resolution of the ventricular repolarization abnormality in synchronization with resolution of her COVID-19 viral illness. *Discussion*s. Although there have been reports of QTc prolongation in COVID-19 patients, previous reports of this are for patients receiving medication that causes QT prolongation. This case uniquely demonstrates the development and resolution of this temporary ventricular repolarization abnormality in a patient with a structurally normal heart with no evidence of myocardial fibrosis or edema on cardiac MRI, that is unexplained by other confounding factors, such as medication. This suggests there may be a direct association between COVID-19 and temporary QTc prolongation.

## 1. Introduction

Prolonged QTc is a potentially dangerous ventricular repolarization disorder that can lead to adverse events including torsades de pointes, ventricular fibrillation cardiac arrest, and sudden cardiac death. Causes include both congenital prolongation and acquired causes. Acquired causes include medication, acute electrolyte disturbances, systemic inflammation, and hypoxia [1–3]. There is increasing evidence that inflammatory cytokines, in particular, interleukin 6 (IL-6), can have a direct effect on ventricular cardiomyocyte ion channels, prolonging the action potential and hence the QTc [4–7]. There are few existing case reports of QTc prolongation in patients with COVID in the absence of conventional risk factors [8, 9]. This case suggests there may be an association with the severe acute respiratory syndrome coronavirus 2 (SARS-CoV-2) virus and temporary QTc prolongation.

## 2. Case Presentation

**Figure 1:**
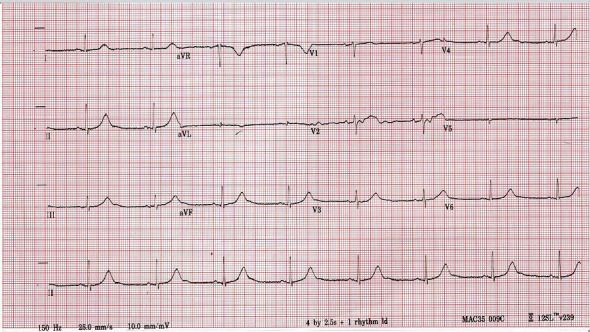
Shows day 1 ECG with a QTc of 495 ms.

We report the case of a 65-year-old Caucasian female referred to hospital by her general practitioner with dyspnoea, chest pain, and general malaise, following a recent diagnosis of COVID-19 in the community two days prior to presenting. She had a 5-day history of dyspnoea on exertion and orthopnoea. This was associated with intermittent pleuritic chest pain and fevers. Her medical history included breast cancer treated with a lumpectomy, chemotherapy, and radiotherapy. There was no family history of sudden cardiac death or sudden adult death syndrome, no family history of long QT syndrome, and no family history of congenital deafness. She was not on any QT-prolonging treatment or any rate-limiting therapy, and her only regular medication was rosuvastatin. Her physical examination on presentation was unremarkable. Laboratory investigations revealed a mildly elevated troponin I of 0.06 ng/mL (normal range 0–0.04 ng/mL) which remained at 0.06 ng/mL on serial checking and a D-dimer of 624 ng/mL. Inflammatory markers were normal on admission with a C-reactive protein (CRP) of 7 mg/L (reference range 0.1–5 mg/L) and a white cell count (WCC) of 3 × 10^9^/L. Her lymphocyte count was 1.25 × 10^9^/L. IL-6 levels were not measured. Radiological investigations on admission revealed a normal chest X-ray, and a CT pulmonary angiogram showed patchy peripheral pulmonary infiltrates with no pulmonary embolus. Her admission ECG showed sinus bradycardia with a heart rate of 47 beats per minute and a QTc of 495 ms, shown in Figure 1. QT intervals and RR intervals were measured manually for the calculation of the QTc using Bazett's method; the calculated values were consistent with the automatic machine measurements.

**Figure 2:**
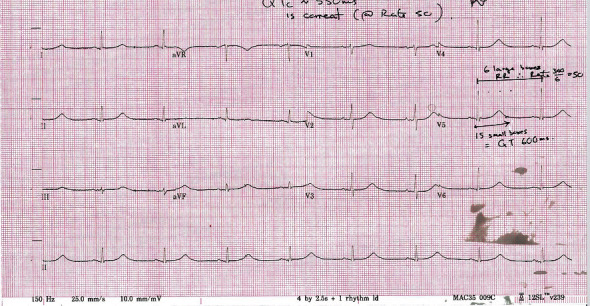
Shows day 2 ECG with a QTc of 621 ms.

**Figure 3:**
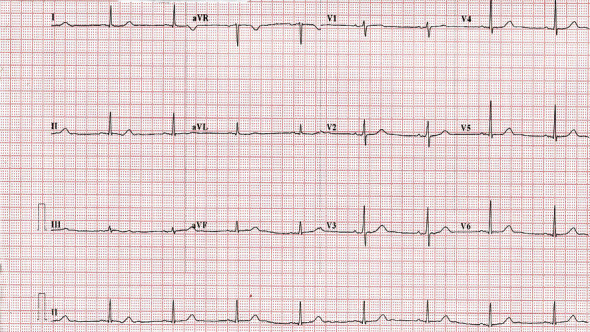
Shows day 24 ECG with a QTc of 445 ms.

A repeat ECG one day postadmission showed a QTc of 621 ms, shown in Figure 2. She was commenced on telemetry with daily monitoring of electrolytes and ECGs. Her serum electrolytes were within normal limits throughout the course of the admission. Medications used throughout the admission included enoxaparin and rosuvastatin regularly and paracetamol, lactulose, and cyclizine as required. Cetirizine was subsequently prescribed after the first week of admission. A cardiac MRI was booked in an external centre, and the patient remained in our hospital until she became noninfectious from COVID-19 prior to transfer for MRI. Her QTc decreased gradually over the course of her 24-day hospital admission. From a respiratory perspective, she remained well, her peripheral oxygen saturation levels were normal throughout the entire admission, and she did not develop any supplementary oxygen requirement. Transthoracic echocardiogram was unremarkable, revealing a structurally normal heart and no evidence of heart failure. Cardiac MRI confirmed a structurally normal heart with no evidence of myocardial fibrosis or edema. A trivial pericardial effusion was present. Her QTc resolved to a normal level of 445 ms, shown in Figure 3. She remained clinically well and was discharged.

## 3. Discussion

The average QTc in healthy persons after puberty is 420 ± 20 milliseconds. Generally, the 99th percentile QTc values are 470 milliseconds in postpubertal males and 480 milliseconds in postpubertal females [10]. A QTc > 500 milliseconds is considered highly abnormal for both males and females. Several manifestations of cardiovascular disease have been previously described in COVID-19 [11]. Existing literature regarding QTc prolongation in COVID-19 focuses on predictable pharmacodynamic effects of offlicense pharmacotherapeutic strategies such as antimalarial drugs [12]. The prolongation previously seen has been attributable to medications with this known effect [13, 14]. This case demonstrates an acute ventricular repolarization abnormality in the setting of COVID-19 infection, that self-resolved as the viral illness course resolved. This self-limiting temporary disturbance, in the absence of alternative explanations, suggests that there may be a direct effect on ventricular repolarization from COVID-19. Cetirizine, an anti-H1 histamine receptor drug, was prescribed following a week of admission, which is recognized to have QT-prolonging potential [15]. It is possible that the effects of COVID-associated systemic inflammation on the QTc may have been enhanced by the concomitant cetirizine treatment.

The relationship between COVID-19 and ventricular repolarization is an area of worldwide interest with an ongoing trial in critically unwell patients to determine the effect of COVID-19 (and its treatments) on ventricular repolarization as measured by the QTc [16]. Systemic inflammation rapidly induces cytokine-mediated ventricular electrical remodeling and significant QTc prolongation [17, 18]. It is possible that an important factor involved in QTc prolongation in COVID-19 is the systemic inflammation characterizing the disease, often described as a “cytokine storm” in which IL-6 seems to play a central role. This systemic inflammation via elevated IL-6 is itself an independent risk factor for QTc prolongation, by modulating cardiomyocyte ion channel expression [18]. Other viral illnesses have been reported to exhibit this effect—HIV seropositivity is associated with a prolonged QT [19]. Physiological changes in acute illness can also lead to a temporary ventricular repolarization abnormality—core body temperature can impact QT duration with moderate hypothermia causing temporary prolongation of the QTc as well as fever [20, 21]. However, in this case, our patient remained normothermic throughout the admission, and her CRP and WCC were only very mildly elevated while these electrocardiographic changes occurred, as shown in Table 1. Although CRP synthesis is largely dependent on direct hepatocyte stimulation by IL-6, it is possible for a mismatch to exist (for example, in patients with abnormal liver function) between circulating levels of IL-6 and CRP. Despite this patient having a normal liver function, it cannot be excluded that the blood concentration of IL-6 (which is the actual mediator of the electrophysiologic effects of systemic inflammation on the cardiomyocyte) was higher than expected based on the CRP levels. However, this case suggests that there may be a novel COVID-19 infection-specific mechanism for QT prolongation not previously described.

## Figures and Tables

**Table 1:** Association between inflammatory markers and corrected QT interval.

	Day 1	Day 2	Day 3	Day 5	Day 6	Day 7	Day 8	Day 10	Day 13	Day 20	Day 24
CRP (mg/L)	7.3	7.4	6.2	9.2	10.4	11	12	15.5	8.2	5.5	Not recorded
WCC (×10^9^/L)	3.57	4.11	2.85	2.97	3.21	2.77	3.7	3.5	5.52	5.44	
QTc (ms)	495	621	561	516	502	479	Not recorded		465	465	445

CRP: C-reactive protein; mg/L: milligrams per liter; WCC: white cell count, QTc: QT interval corrected; ms: milliseconds.

## Data Availability

Background information is available on medical database Pubmed.
